# Chronic Pain: Among Tertiary Care Psychiatric Out-Patients in Singapore—Prevalence and Associations with Psychiatric Disorders

**DOI:** 10.1155/2022/1825132

**Published:** 2022-04-15

**Authors:** Pratika Satghare, Edimansyah Bin Abdin, Aditi Hombali, Wen Lin Teh, Ellaisha Samari, Boon Yiang Chua, Swapna Verma, Yee Ming Mok, Siow Ann Chong, Mythily Subramaniam

**Affiliations:** ^1^Research Department, Institute of Mental Health, Singapore; ^2^Department of Early Psychosis, Institute of Mental Health, Singapore; ^3^Department of Mood and Anxiety, Institute of Mental Health, Singapore

## Abstract

**Objective:**

The study aimed to determine the prevalence and severity of chronic pain and its associations amongst psychiatric out-patients in a tertiary care hospital in Singapore. *Methodology*. The cross-sectional study was conducted among 290 psychiatric out-patients aged 21–65 years. Sociodemographic and clinical information, as well as data from Brief Pain Inventory-Short Form (BPI-sf), Beck's Depression Inventory II (BDI-II), and Beck's Anxiety Inventory (BAI) were collected. Cut points (C.P.s) dividing the sample into mild, moderate, and severe groups were created for the ratings of average pain. Eight possible cut-off values for the C.P.s between 3 and 7, representing 8 different categorical variables, were created and their relationships were examined with BPI's set of seven interference items using multivariate analysis of variance. Sociodemographic and clinical correlates of chronic pain were determined using multinomial logistic regression analysis. Analysis of covariance was used to determine the association of BPI with continuous scores of BAI and BDI.

**Results:**

Based on the C.P. pain severity classification, 38.5% of the sample had mild pain, 22.9% had moderate pain, and 11.8% had severe pain. Patients with severe pain were more likely to be associated with older age (*p* ≤ 0.006) (versus young age), less likely to be married (*p* ≤ 0.025) (versus single), and more likely to have high risk for obesity (*p* ≤ 0.030) (versus low risk for obesity). Participants with mild pain were seen to be significantly associated with older age (*p* ≤ 0.021), whereas moderate pain (*p* ≤ 0.002) and severe pain (*p* ≤ 0.001) (versus no pain) were seen to be significantly associated with higher BAI scores.

**Conclusion:**

The current study observed high prevalence of pain among patients with psychiatric illness that was determined by optimal C.P.s for mild, moderate, and severe pain. Patients diagnosed with anxiety disorders and those with higher BMI were seen to be associated with pain of moderate to severe intensity. Improving the knowledge of correlates and co-morbidities of physical pain would aid in early identification, use of prophylactic strategies, and the intervention techniques to formulate basic guidelines for pain management among psychiatric population.

## 1. Introduction

Pain or “poena” in Latin means suffering. Pain is an unpleasant physical sensation caused by an illness or injury that can range from mild, localized discomfort to agony. Pain is one of the most common problems globally and is one of the main reasons for seeking medical help [1]. Aside from the need for diagnostic evaluation and symptom relief, people seek medical help because pain interferes with daily activities causes worry and emotional distress, undermines confidence in one's health, and affects the quality of life [2]. In addition, psychological health and performance of responsibilities in work and family life are also often significantly impaired due to pain [3].

Chronic pain is one of the most pervasive symptoms in the community and primary care setting [4, 5]. People suffer from physical pain in the presence or absence of any past trauma or injury or evidence of any body damage. World Health Organization (WHO) has reaffirmed the prominence of chronic pain as an important co-morbidity associated with the four leading contributors of global burden of diseases, i.e., unipolar depression, coronary heart disease, cerebrovascular disease, and road traffic accidents [6]. However, chronic pain is much more than just co-morbidity with any other identifiable disease. Not only can pain be a symptom of other underlying physical conditions, but it also can be a symptom of primary disease, characterized by changes in the central nervous system such as painful diabetic neuropathy [6]. World Health Organization (WHO) in its latest edition of International Classification of Diseases (ICD) has included chronic pain and its classification in ICD's category of ‘Chronic Pain' for the first time, which is based on the biopsychosocial model [7]. Research has shown that chronic secondary pain is more common among individuals with other underlying disease conditions than chronic primary pain [7, 8].

Definition of chronic pain varies, ranging from pain involving no arbitrarily fixed durations but extending beyond the expected period of healing to recurring, lingering pain present for at least three months, among different studies depending on the study design and population [9, 10]. A cross-sectional Internet-based survey observed a considerable burden of 30.7% of chronic, recurrent, or persistent pain lasting for at least 6 months among U.S. adults [11]. A multicentric study among primary care patients examined the prevalence of persistent pain, defined as ‘physical pain present for 6 months or more during the previous year'. The prevalence of persistent pain in Greece (12%), Italy (13%), England (20%), Japan (12%), China (13%), Brazil (31%), and Chile (33%) was high and associated with a marked reduction in indicators of well-being, especially psychological illness and self-rated health status [12, 13].

Chronic physical pain interacts with psychiatric symptoms and psychiatric disorders such as fatigue, irritability, hopelessness, depression, and anxiety in a complex manner. It is frequently comorbid with psychiatric disorders that influence it [14]. Significant positive associations were observed between chronic pain and anxiety disorders [15]. Prior epidemiological literature suggests chronic pain to be strongly associated with depressive disorders. The characteristics that strongly predict depression are diffuseness of pain and the extent to which pain interferes with activity and other symptoms like sleep problems, reduced productivity, and social relationships [2]. In a health maintenance organization, employees who reported at least one pain condition had more symptoms of depression and anxiety than individuals without any pain condition [16]. Birgenheir et al.'s study noted that the presence of chronic pain is one of the potential barriers in the recovery process of psychiatric illnesses, making it difficult to treat them and worsening their prognosis [17]. A bidirectional relationship exists between chronic pain and psychiatric disorders [18]. Being interconnected by neurobiological pathways, the co-occurrence of depression and physical pain adversely influences the other and is often observed as a negative impact on physical and emotional quality of life [16, 18].

The prevalence estimates of chronic physical pain noted by prior cross-sectional studies among Singapore adults ranged from 7 to 8.7% [2, 19] while that among older adults was 19.5% [20]. However, there is a dearth of knowledge on the prevalence of chronic physical pain in patients with psychiatric disorders and the sociodemographic correlates associated with it. The current study thus aims to establish the prevalence of chronic physical pain among out - patients with anxiety disorders, depressive disorders or schizophrenia spectrum disorders in Singapore and explore its associations with sociodemographic factors, body mass index (BMI) status, and clinical correlates.

## 2. Methodology

### 2.1. Sample, Setting, Procedure

This cross-sectional study was conducted among out-patients seeking treatment in a tertiary care psychiatric hospital, Institute of Mental Health (IMH) Singapore. The highest workload of patients comprises those with a diagnosis of schizophrenia spectrum disorders, depressive and anxiety disorders in IMH. Hence, out-patients aged 21–65 years, fluent in English, and diagnosed with anxiety disorders, depressive disorders, or schizophrenia spectrum disorders were recruited for this study. Participants unable to read and understand English and with any intellectual disability were excluded from the study. Participants were recruited through referrals from clinicians. The inclusion criteria and study details were also disseminated using flyers and posters to allow interested participants to contact the research team directly. The total number of participants approached for the study was not captured. Prior to enrolment, study team members explained the details of the study, its importance, benefits, and risks, and emphasized its voluntary and confidential nature to potential participants. Written informed consent was obtained from eligible participants. Information related to the primary psychiatric illness of the participants was obtained from their electronic medical records upon receiving consent. Following this, the participants completed the self-administered survey. Participants were reimbursed 30 Singapore dollars (SGD) on completion of the study for their time.

The ethics committee of the National Healthcare Group-Domain Specific Review Board Singapore approved the study (DSRB No. 2016/01159).

### 2.2. Questionnaires

The survey comprised the following questionnaires:

1. Sociodemographic questions: these included age, gender, ethnicity, education level, marital status, employment status, and personal/household income. The weight and height of the participants were captured from the medical records.
2. Clinical data: information related to only primary psychiatric illness of the participants, excluding any medication history was obtained from their electronic medical records.
3. Brief Pain Inventory—Short Form (BPI-sf) [21]:it is a 9 item self-administered questionnaire used to evaluate the severity of pain experienced (sensory dimension) by patients and its impact (reactive dimension) on their daily functioning. The BPI has also demonstrated both reliability and validity across cultures and languages and has been adopted in many countries for clinical pain assessment, epidemiological studies, and in studies examining the effectiveness of pain treatment [22]. Cronbach alpha reliability ranges from 0.77 to 0.91 across studies [21, 23]. The BPI had a high internal consistency in our sample both in the pain severity (Cronbach's *α* = 0.94) and interference (Cronbach's *α* = 0.96) factors.
4. Body Mass Index (BMI): nutritional status was assessed by means of the body mass index [(BMI = body mass/(height)^2^]. Participants' BMI was calculated using an ultrasonic height sensor and load cell (Avamech, model B1000). The BMI scores were categorized according to cut-off points established by Obesity and Metabolic Unit in the Singapore General Hospital (2016): those having BMI score of 27.5 and above as high risk for obesity, score of 23–27.4 as moderate risk for obesity, 18.5–22.9 as low risk for obesity, and scores below 18.5 as risk for nutritional deficiency.
5. Beck's Depression Inventory II (BDI-II) [24]: it is a 21 item self-report questionnaire that measures the severity of depression having high internal consistency (*α* = 0.91) [25]. A high internal consistency was similarly found in current sample (Cronbach's *α* = 0.96).
6. Beck's Anxiety Inventory (BAI) [26]: this is a 21-item questionnaire measuring the severity of anxiety in adults and adolescents having a high level of internal consistency (*α* = 0.94) [27]. A high internal consistency was similarly observed in current sample (Cronbach's *α* = 0.95).
7. Chronic medical condition checklist [28]: this is an interviewer administered checklist used to measure the chronic physical health conditions of the respondents. The respondents were asked to report any of the disorders listed in the checklist. The list comprised chronic medical conditions that are prevalent in Singapore and included asthma, high blood sugar or diabetes, hypertension or high blood pressure, arthritis or rheumatism, cancer, neurological conditions such as epilepsy or convulsions, Parkinson's disease, stroke or major paralysis-inability to use arms or walk, congestive heart failure (including a heart attack, coronary heart disease, angina or any other heart disease), back problems including disc or spine, stomach ulcer, chronic inflamed bowel, enteritis or colitis, thyroid disease, kidney failure, chronic lung disease such as chronic bronchitis or emphysema (excluding asthma), and high cholesterol or hyperlipidemia. Details of the age of diagnosis and treatment history were recorded for participants who had been diagnosed with a medical condition.

### 2.3. Sample Size

The sample size was calculated using single proportion formula to produce a precise estimate with a margin of error equal to 5%. Based on the prevalence of chronic pain in adult psychiatric patients of 18% as reported by prior research [29], an effective sample size of 290 patients was calculated after adjusting for incomplete returns, to estimate the prevalence of pain among psychiatric out-patient population.

### 2.4. Data Analysis

Descriptive analyses were conducted to describe the characteristics of the study sample, with means and standard deviations (S.D.) being calculated for continuous variables and frequency and percentage for categorical variables. Cut points (C.P.s) that divided the sample into mild, moderate, and severe groups were created based on the ratings of average pain (BPI Item 5) using Serlin et al.‘s analytical approach [30]. Only average pain cut points were used in our analysis because average pain rating is more representative of chronic physical pain among psychiatric out-patients [31]. In order to establish ‘optimal' C.P.s for mild, moderate, and severe pain by each diagnosis, pain severity categorized according to the C.P.s was treated as an independent variable. Seven interference items (items 9a to 9g) from the BPI were dependent variables. Thus, 8 different binary variables were created to represent 8 possible cut-off values for the C.P.s between 3 and 7. The relationship of these 8 variables with BPI's set of seven interference items was examined using multivariate analysis of variance (MANOVA). The criteria used to determine the optimal set of C.P.s for mild, moderate, and severe pain was based on MANOVA tests. Similar criterion based on MANOVA test has been used previously by Serlin et al. (1995) [30]. MANOVA test that yielded the largest F ratio for the between category effect on the 7 interference items as indicated by Pillai's trace, Wilk's lambda, and Hotellings trace F statistics and should be consistent across the diagnosis [30]. The lowest median rank of the ranking of these three statistics was used to determine the optimal C.P.s.

Sociodemographic correlates of chronic pain and its association with BMI were determined using multinomial logistic regression analysis. Analysis of covariance (ANCOVA) was used to determine the association of BPI using different C.P.s for grading of average pain for interference items as dependent scores. A *p* value of ≤0.05 was considered statistically significant using two-sided tests. For the ANOVA analyses, the *p* value presented for each pairwise contrast was adjusted so that a value of ≤0.05 indicates statistical significance. Data were analysed using SPSS for Windows version 22.0.

## 3. Results

### 3.1. Participant Characteristics

The current study recruited 290 out-patients, seeking treatment in a tertiary care psychiatric hospital in Singapore. The sample was approximately equally distributed in terms of gender, with males comprising 49% of the sample. Majority of participants belonged to Chinese ethnicity (60%) followed by Malays (20%), Indians (16.2%), and other ethnic groups (3.8%). Of the total participants recruited, 35.2% had a diagnosis of schizophrenia spectrum disorders, 33.8% had depressive disorders, and 31% had anxiety disorders (Table 1).

The mean age of study participants was 39.6 years (SD = 11.6 years). The BDI-II and BAI scores averaged to 19.6 (SD = 16.1) and 15.9 (SD = 13.8), respectively, in the current study sample, whereas mean of BMI was 26.9 (SD = 6.2). About 37.6% of patients suffered from painful chronic conditions. The percentage of painful chronic conditions were significantly different (*p* ≤ 0.022) among patients diagnosed with anxiety disorders (26.7%), depressive disorders (45.9%), and schizophrenia spectrum disorders (39.2%).

### 3.2. To Establish Cut Point (C.P.) according to Disorders and Prevalence (s)

The analysis was done to select best optimal C.P. that is sensitive to the condition of the participants. The analysis resulted in different optimal C.P.s for out-patients with anxiety disorders, depressive disorders, and schizophrenia spectrum disorders. For average pain among the overall sample, the optimal C.P.s were (4, 6) (1 to 4 is mild pain, > 4 to 6 is moderate pain, and >6 to 10 is severe pain) because they had the lowest median rank of the ranked between-Category-F-ratios, using Pillai's trace, Wilk's lambda, and Hotelling's trace statistics (Table 2). Also, same optimal C.P.s (4, 6) were observed for patients with anxiety and depressive disorders for average pain (Tables 3 and 4). For average pain among patients with schizophrenia spectrum disorders, the optimal C.P.s were (3, 5) (1 to 3 is mild pain, >3 to 5 is moderate pain, and >5 to 10 is severe pain) (Table 5). Ranks for C.P. sets from the multivariate analysis of variance to determine optimal C.P.s using average pain intensity scores and the interference items from the Brief Pain Inventory (BPI) among patients with psychiatric illnesses (Tables 2–5) are available in the supplementary document. With the pain severity based C.P. classification, 38.5% of the sample had mild pain, 22.9% had moderate pain, and 11.8% had severe pain, respectively (Table 1).

Ranks for cut point (C.P.) sets from the multivariate analysis of variance to determine optimal C.P.s using average pain intensity scores and the interference items from the Brief Pain Inventory (BPI) among patients with psychiatric illnesses (Tables 2–5).

Interference as per diagnosis is shown in Tables 3–5.

### 3.3. Sociodemographic and Clinical Characteristics according to Pain Severity Rating with the Optimal Cut Points (C.P.s)

On comparing the three pain severity groups with the “no pain” group, no differences were found in gender, ethnicity, and education level completed. However, patients in the mild (*p* ≤ 0.02) and severe (*p* ≤ 0.006) pain group were more likely to be of older age group, and less likely to be married (*p* ≤ 0.025) (Table 6). For pain severity rating, participants with higher BAI scores were seen to be significantly associated with moderate (*p* ≤ 0.002) and severe pain (*p* ≤ 0.001) (versus no pain) (Table 6). High risk for obesity was seen to be significantly associated with severe pain (*p* ≤ 0.030) (versus no pain) (Table 6).

## 4. Discussion

The current cross-sectional study established that optimal C.P.s for average pain differed between participants diagnosed with schizophrenia spectrum disorder, depression and, anxiety disorders. The overall prevalence of pain was 73.3% among psychiatric out-patients. 37.6% of patients suffered from painful chronic conditions. Other noteworthy findings regarding pain severity rating were that moderate and severe pain were significantly associated with higher BAI scores while severe pain was associated with high risk for obesity.

The finding of different C.P.s of chronic physical pain for patients with schizophrenia spectrum disorders, anxiety and depressive disorders is novel to the current study. The C.P.s established in current study were (3, 5) for those with the diagnosis of schizophrenia spectrum disorder and (4, 6) for patients diagnosed with depressive and anxiety disorders respectively. The lower C.P.s among patients with Schizophrenia spectrum disorders for an average pain intensity in our study indicate that patients with diagnosis of Schizophrenia spectrum disorder may have high levels of interference as an outcome to average pain. It could be because average pain might be perceived by patients with schizophrenia spectrum disorders differently as compared to depressive and anxiety disorders. Contrary to our findings, psychotic analgesia may occur among patients with schizophrenia spectrum disorder due to the presence of severe negative symptoms and cognitive impairment. Outcome of which is often observed as deficit in identification, and categorization of pain, reduced level of emotional awareness to painful stimuli resulting in less willingness to verbalise pain [32, 33]. On the other hand, maladaptive coping skills among patients with anxiety disorders may lead to self-perpetuating cycle that stimulates and maintains increased emotional reactivity, impairing the ability to modulate pain [34]. This may further amplify fear and severity of anxiety symptoms resulting in heightened attention towards painful stimuli leading to increased pain intensity [32, 35]. Similarly, an interaction between depressive disorder and pain; known as depression-pain dyad or depression pain syndrome, imply that these conditions, as they share analogous pathophysiological pathways and neurotransmitters often co-exist and exacerbate one another [18, 36]. They often exhibit directly proportional relationship; patients seeking treatment for depressive disorder experience frequent episodes of pain symptoms with increased pain chronicity [36].

Previous literature has found that the prevalence of pain among patients with psychiatric illnesses ranges from 15% to 65% [29, 37]. A higher prevalence of pain (73.3%) was noted in the current study conducted among psychiatric out-patients. The differences in the prevalence estimates of pain can be partly due to the diverse research methods incorporated by the studies, definitions used for chronic pain, sample selection procedures, and assessments carried out [38]. In addition to this, the study population's sociodemographic construct and their cultural differences in coping strategies are seen to influence prevalence rates considerably [12]. Cultural attitudes and religious beliefs have vital influences on developing an individual's pattern of experiencing, responding, and expressing pain, for instance, some cultures encourage expression of pain and make it public, whereas others may not communicate keeping it private [39].

The current study found that older adults were more likely to suffer from mild to severe pain. This observation reflects the findings documented by prior population studies that pain severity increases with age [38, 40]. This could be attributed to existing psychiatric disorder, longer pain duration, aging process and the fact that being economically active, they are more likely to report the pain complaints which may cause working impairments and disabilities [41]. The current study noted that married participants were less likely to report chronic pain which mirrors findings from prior research. Living with a spouse or partner in a happy union may strengthen the patient's ability to sustain a sense of pain coping efficacy during pain episodes due to patient's perceptions that their spouse's support is highly responsive to their needs as seen in prior research demonstrating use of spouse assisted pain coping skills training [42] and also empathic partner communications as compared to patients who are single [43]. Another possible explanation for this could be the prevailing model which emphasises influence of spouse on patient's functioning by aiding adaptive and limiting maladaptive responses to pain exacerbations [42].

Our findings, in line with prior literature, noted that participants with higher BAI scores were associated with moderate to severe pain. This association between severity of anxiety symptoms (i.e., High BAI scores) and physical pain tends to increase the suffering associated with pain, which has been elucidated on many levels [44]. Pain is an adaptive process, a warning signal, that leads the nervous system to be in a persistent state of reactivity or central sensitization and is associated with physiological arousal, similar to anxiety [45]. But among patients with pre-existing anxiety symptoms and comorbid physical pain, there is loss of this adaptive quality. Such loss results in detrimental physical effects of prolonged physiological arousal due to pain and individual's response to pain is misinterpreted [6, 46]. Prior research has endorsed that presence of higher BAI scores with comorbid pain can increase pain ratings by changing its threshold and tolerance [47, 48]. However, no significant association was observed between schizophrenia spectrum disorders and chronic pain in the current study. The explanation for this may be that in patients with psychosis, especially among those with schizophrenia, usually a negative association with symptoms of pain is observed; but pain when present, is inexplicable or acquires delusional quality and may rarely occur as the presenting complaint [32].

Obesity and chronic pain are seen to impact each other adversely and their association can be explained as a cause, consequence or related in a reciprocal manner [49]. The current study noted that participants with high risk for obesity were more likely to experience pain of severe intensity as compared to participants with normal body weight. This could be attributed to the fact that presence of pain symptoms of severe intensity may interfere with daily functioning and inhibit physical activity resulting in a sedentary lifestyle [50]. In addition to this, functional limitations may cause frustration, distress, fear of movement, lack of motivation and poor self-esteem as commonly seen among patients with psychiatric illness such as depressive disorder which may promote emotional eating or eating food items that represent comfort [51–53]. Such behaviour in addition to poor physical activity due to pain symptoms is a risk factor for obesity [50]. Whereas on the other hand, endocrinal changes are seen to be associated with obesity which cause chronic systemic inflammation in body giving rise to physical pain symptoms by altering pain modulation mechanism [49].

Certain limitations need to be considered while interpreting the study findings. Since the patient's participation in the study was voluntary, the reasons for refusal to participate and ineligibility were not captured. Also, patients who refused to participate in this study may have higher or lower pain intensity scores. There is a possibility of selection bias because of convenience sampling of English-speaking psychiatric out-patients. As the questionnaires were available only in English language, we recruited only those who could read and understand English language. Unfortunately, we are unable to provide accurate numbers of patients who could not speak English. In line with findings from previous reports, the current study also concentrated on a range from (3, 5) to (5, 7) but did not consider the full range of possible C.P.s. Another limitation was that being a cross-sectional study, we were unable to determine any causal relationships between physical pain and depressive or anxiety symptoms. The study was conducted in a single, tertiary care psychiatric hospital, among out-patients aged 21–65 years, and hence the results may not be generalizable to other clinical settings.

Notwithstanding the limitations, the strengths of the study include it being a single-site study with large sample size, conducted by trained researchers using structured instruments administered in a multiethnic Asian population with psychiatric illnesses. To our knowledge, this is the first study that has determined the optimal C.P.s that categorize BPI pain scores as mild, moderate, and severe pain, based on patient's ratings of average physical pain and to provide optimal C.P.s for depressive, anxiety, and schizophrenia spectrum disorders among psychiatric out-patients. This pain score categorization may be beneficial to communicate and interpret the scores to patients with different psychiatric disorders. The study findings add to the growing literature on prevalence of chronic pain among patients with psychiatric disorders and improves the knowledge on its relative associations in psychiatric population.

## 5. Conclusion and Implications

The present study established a high prevalence of chronic physical pain among patients with psychiatric illness that was determined by optimal C.P.s for mild, moderate, and severe pain intensity. Psychiatric illness with comorbid pain significantly influences the course of the former condition and worsens its prognosis. The high prevalence of pain in the current study sample thus reinforces the need for interaction between various areas of clinical expertise to ensure systematic assessment and best pain management along with treatment of psychiatric disorder, by focusing on the psychosocial and psychological aspects of pain conditions.

The C.P.s for pain intensity established in the current study can be utilised as a screening instrument to detect pain, and the different cut-off point scores for different conditions can be considered when measuring pain among patients with psychiatric illness. In addition to this, improving the knowledge on correlates and co-morbidities of physical pain would aid in early identification, use of prophylactic strategies, and the intervention techniques may assist in formulating guidelines for pain management among psychiatric population. This could be achieved by engaging mental health clinicians in addressing pain treatment as they are in best position for assessment of pain, introduce and initiate customised treatment plans, suggest behavioural treatment according to patient's condition, as well as encourage them to participate in self-management and physical activities. This in turn, would ease individual suffering, lessen societal expenses, and lower the global burden associated with chronic pain conditions.

## Figures and Tables

**Table 1:** Descriptive statistics of sociodemographic and clinical characteristics of the study sample.

Variable	N	Percentage	Mean	SD
*Age (years)*			39.6	11.6
21–40 years	151	52.1		
41–65 years	139	47.9		
*Gender*				
Male	142	49		
Female	148	51		
*Ethnicity*				
Chinese	174	60		
Malay	58	20		
Indian	47	16.2		
Others	11	3.8		
*Marital status*				
Single	173	59.7		
Married	71	24.5		
Widowed/separated/divorced	46	15.9		
*Education*				
Secondary or below	20	6.9		
Pre-university	89	30.7		
Tertiary or above	181	62.4		
*Diagnosis*				
Depressive disorders	98	33.8		
Anxiety disorder	90	31		
Schizophrenia spectrum disorder	102	35.2		
*Pain severity*				
None	77	26.7		
Mild	111	38.5		
Moderate	66	22.9		
Severe	34	11.8		
*Painful chronic conditions* ^*∗*^				
No	181	62.4		
Yes	109	37.6		
*BDI-II scores*			19.6	16.1
None	121	41.7	11.8	13.3
Mild	48	16.6	18.9	15.7
Moderate	45	15.5	26.1	15.1
Severe	76	26.2	26.2	18
*BAI scores*			15.9	13.8
None	101	34.8	9.1	9.4
Mild	68	23.45	13.5	11.1
Moderate	53	18.3	22.3	14.3
Severe	68	23.5	27	18
*BMI*			26.9	6.2
Low risk for obesity	89	30.8		
Risk for nutritional deficiency	118	40.83		
Moderate risk for obesity	68	23.53		
High risk for obesity	14	4.84		
*Chronic medical condition*				
No	103	35.52		
Yes	187	64.48		

^*∗*^Painful chronic conditions-arthritis or rheumatism, cancer, back problems including disc or spine, stomach ulcer, chronic inflamed bowel, enteritis or colitis, diabetic neuralgia, musculoskeletal pain (hypothyroidism), etc.

**Table 2:** Overall interference.

Cut- points	Wilks' lambda		Pillai's trace		Hotelling's trace		Median rank
	Rank	F	Rank	F	Rank	F	
CPA 3,5	4	10.38	4	9.13	5	11.66	4
CPA 3,6	2	11.32	2	10.15	2	12.51	2
CPA 3,7	5	10.25	6	9	6	11.53	6
CPA 4,5	4	10.38	5	9.05	4	11.75	5
CPA 4,6	1	11.62	1	10.25	1	13.02	1
CPA 4,7	3	10.89	3	9.42	3	12.41	3
CPA 5,6	6	6.77	7	6.18	7	7.36	7
CPA 5,7	7	6.59	8	5.98	8	7.2	8

**Table 3:** Anxiety disorders.

Cut points	Wilk's lambda		Pillai's trace		Hotelling's trace		Median rank
	Rank	F	Rank	F	Rank	F	
CPA 3,5	6	4.08	6	3.43	6	4.74	6
CPA 3,6	3	4.72	3	4.18	3	5.26	3
CPA 3,7	4	4.41	4	3.97	4	4.85	4
CPA 4,5	5	4.27	5	3.71	5	4.83	5
CPA 4,6	1	5.05	1	4.56	1	5.53	1
CPA 4,7	2	4.82	2	4.23	2	5.39	2
CPA 5,6	8	3.02	8	2.75	8	3.29	8
CPA 5,7	7	3.22	7	3.04	7	3.4	7

**Table 4:** Depressive disorders.

Cut points	Wilks' lambda		Pillai's trace		Hotelling's trace		Median rank
	Rank	F	Rank	F	Rank	F	
CPA 3,5	5	4.63	5	4.08	5	5.19	5
CPA 3,6	3	5.72	2	4.94	4	6.53	3
CPA 3,7	6	4.04	6	3.67	7	4.42	6
CPA 4,5	2	5.76	3	4.9	3	6.65	2
CPA 4,6	1	7.12	1	5.9	1	8.42	1
CPA 4,7	4	5.69	4	4.78	3	6.65	4
CPA 5,6	7	4.02	7	3.31	6	4.75	7
CPA 5,7	8	3.38	8	2.91	2	7.67	8

**Table 5:** Schizophrenia spectrum disorders.

Cut points	Wilks' lambda		Pillai's trace		Hotelling's trace		Median rank
	Rank	F	Rank	F	Rank	F	
CPA 3,5	1	3.3	1	3.04	1	3.56	1
CPA 3,6	2	3.25	1	3.04	2	3.48	2
CPA 3,7	3	2.85	2	2.47	3	3.23	3
CPA 4,5	4	2.59	3	2.45	4	2.73	4
CPA 4,6	5	2.56	4	2.44	5	2.68	5
CPA 4,7	6	2.2	5	2	6	2.4	6
CPA 5,6	8	1.69	6	1.62	8	1.75	8
CPA 5,7	7	1.72	7	1.67	7	1.78	7

**Table 6:** Multinomial logistic regression showing sociodemographic factors and clinical correlates.

Sociodemographic/clinical variables	Pain severity																	
	Mild						Moderate						Severe					
	OR		*p* value		95% CI		OR		*p* value		95% CI		OR		*p* value		95% CI	
*Age*																		
21–40	Ref		Ref		Ref		Ref		Ref		Ref		Ref		Ref		Ref	
41–65	2.65		**0.02**		1.16–6.09		2.42		0.07		0.91–6.41		6.29		**0.006**		1.68–23.55	
*Gender*																		
Male	Ref		Ref		Ref		Ref		Ref		Ref		Ref		Ref		Ref	
Female	1.07		0.96		0.50–2.07		0.93		0.92		0.41–2.23		0.46		0.19		0.14–1.46	
*Ethnicity*																		
Chinese		Ref		Ref		Ref		Ref		Ref		Ref		Ref		Ref		Ref
Malay		0.97		0.87		0.35–2.36		0.85		0.78		0.27–2.64		2.82		0.14		0.70–11.24
Indian		0.8		0.67		0.28–2.25		1.27		0.68		0.40–3.99		1.96		0.37		0.44–8.57
Others		0.76		0.76		0.13–4.41		0.34		0.41		0.02–4.61		0.64		0.77		0.03–13.70
*Education*																		
Pre-university		Ref		Ref		Ref		Ref		Ref		Ref		Ref		Ref		Ref
Secondary and below		1.09		0.9		0.22–5.21		0.65		0.69		0.08–5.07		2.32		0.39		0.02–0.77
Tertiary and above		0.62		0.2		0.27–1.41		1.63		0.29		0.65–4.03		1.08		0.9		0.30–3.80
*Marital status*																		
Single		Ref		Ref		Ref		Ref		Ref		Ref		Ref		Ref		Ref
Married		0.59		0.24		0.25–1.41		0.59		0.33		0.20–1.70		0.12		**0.02**		0.02–0.77
Widow/separated/divorced		0.44		0.17		0.14–1.41		0.85		0.81		0.24–3.06		1.03		0.96		0.22–4.79
*BMI*																		
Low risk for obesity		Ref		Ref		Ref		Ref		Ref		Ref		Ref		Ref		Ref
Risk for nutritional deficiency		1.99		0.1		0.85–4.63		2.03		0.17		0.72–5.74		4.37		0.05		0.97–19.60
Moderate risk for obesity		0.62		0.31		0.24–1.56		1.41		0.52		0.48–4.11		2.05		0.37		0.41–10.21
High risk for obesity		3.24		0.32		0.32–32.74		5.41		0.18		0.45–64.82		23.66		**0.03**		1.35–412.91
*BDI*		1.03		0.07		0.99–1.06		1.02		0.12		0.99–1.06		0.99		0.86		0.94–1.04
*BAI*		1.02		0.28		0.98–1.07		1.07		**0.002**		1.02–1.13		1.12		**0.001**		1.05–1.19
*Any chronic condition*		1.53		0.23		0.76–3.10		1.6		0.27		1.02–1.13		2.13		0.22		0.63–7.22

Bold values are significant *p* values.

## Data Availability

Data used to support the findings of this study are included within the article.
